# Single-Cell Transcriptomics Reveals the Cellular Heterogeneity of Cardiovascular Diseases

**DOI:** 10.3389/fcvm.2021.643519

**Published:** 2021-06-11

**Authors:** Mengxia Fu, Jiangping Song

**Affiliations:** ^1^State Key Laboratory of Cardiovascular Disease, Fuwai Hospital, National Center for Cardiovascular Diseases, Chinese Academy of Medical Sciences and Peking Union Medical College, Beijing, China; ^2^The Cardiomyopathy Research Group at Fuwai Hospital, Beijing, China; ^3^Department of Cardiovascular Surgery, Fuwai Hospital, National Center for Cardiovascular Diseases, Chinese Academy of Medical Sciences and Peking Union Medical College, Beijing, China

**Keywords:** heterogeneity, vasculature, aortic aneurysm, atherosclerosis, pulmonary hypertension, single-cell transcriptomics

## Abstract

“*A world in a wild flower, and a bodhi in a leaf*,” small cells contain huge secrets. The vasculature is composed of many multifunctional cell subpopulations, each of which is involved in the occurrence and development of cardiovascular diseases. Single-cell transcriptomics captures the full picture of genes expressed within individual cells, identifies rare or *de novo* cell subpopulations, analyzes single-cell trajectory and stem cell or progenitor cell lineage conversion, and compares healthy tissue and disease-related tissue at single-cell resolution. Single-cell transcriptomics has had a profound effect on the field of cardiovascular research over the past decade, as evidenced by the construction of cardiovascular cell landscape, as well as the clarification of cardiovascular diseases and the mechanism of stem cell or progenitor cell differentiation. The classification and proportion of cell subpopulations in vasculature vary with species, location, genotype, and disease, exhibiting unique gene expression characteristics in organ development, disease progression, and regression. Specific gene markers are expected to be the diagnostic criteria, therapeutic targets, or prognostic indicators of diseases. Therefore, treatment of vascular disease still has lots of potentials to develop. Herein, we summarize the cell clusters and gene expression patterns in normal vasculature and atherosclerosis, aortic aneurysm, and pulmonary hypertension to reveal vascular heterogeneity and new regulatory factors of cardiovascular disease in the use of single-cell transcriptomics and discuss its current limitations and promising clinical potential.

## Introduction

### Vasculature

The vascular system is a closed circulatory system composed of arteries, veins, and capillaries. It is distributed in various organs of the body, responsible for transporting gas, nutrients, metabolites, and various cells, providing paracrine signal molecules to surrounding tissues, and participating in a whole set of physiological activities from tissue development and remodeling to metabolism and inflammation (1, 2).

The different origins of germ layer, micromorphology, and classical markers of the endothelial cells (ECs), vascular smooth muscle cells (VSMCs), fibroblasts, immune cells, mesenchymal stem cells, and pericytes allow each to perform their function correctly. ECs form the intima of blood vessels and direct contact with the cells and molecular components of blood. They are not only barriers but also endocrine cells, controlling the relaxation and contraction of blood vessels and blood coagulation. VSMCs form the media of blood vessels, providing structural support and participating in the regulation of vascular tension and blood pressure. Fibroblasts are responsible for depositing extracellular matrix around blood vessels and constructing fiber networks. Immune cells come from circulating blood and vascular adventitia to resist pathogen erosion and maintain vascular health. Mesenchymal stem cells exist in the adventitia of blood vessels and have the ability of multidirectional differentiation. Under pathological conditions, different types of cells and different functional cell subpopulations are involved in the occurrence and development of vascular diseases.

### Major Cardiovascular Diseases

Coronary atherosclerosis is the most common cardiovascular disease, which is characterized by chronic inflammation of arteries and formation and accumulation of plaque in the intimal layer. Aortic aneurysm (AA) is a common cardiovascular disease; the pathological features of AA include low-grade inflammation of aortic wall, degradation of extracellular matrix (ECM), and progressive vascular smooth muscle cell (VSMC) degeneration (3). Pulmonary hypertension (PH) is common and characterized by inflammation, pulmonary vascular remodeling, and angio-obliteration (4).

Clinical complications of atherosclerosis, aortic aneurysm, and pulmonary hypertension are the main causes of death worldwide (5–8), hence, a more focused approach is required. As a variety of cell types are involved and/or dysfunctional, a completely effective treatment strategy has not been developed. Attributing the progression of the lesion to a specific cell type is challenging, because of the high heterogeneity of cells not only within but also around the lesion, and single-cell transcriptomics hold a potential in understanding cellular functions, which plays a significant role in contributing to medical science and transcriptomics advancement.

### Single-Cell Transcriptomics

Single-cell omics includes single-cell genomics, single-cell transcriptomics, single-cell epigenomics, and single-cell proteomics. It combines high-throughput omics technology and traditional single-cell research methods to solve the problem of throughput and resolution, enables a comprehensive analysis of cells, and reveals new cell subpopulations and cell interactions.

The widely used single-cell omics technique is single-cell transcriptome sequencing technology. Traditional sequencing methods are performed on tissue samples or cell populations, in which case biological differences between cells may be masked by averaging or mistaken for technical noise. However, each cell is unique—it occupies a unique place in space, carries obvious errors in its replicated genome, and is affected by programmed and induced changes in gene expression. Thus, single-cell transcriptome sequencing is essential for studying single cells. Single-cell transcriptome sequencing enables researchers to zoom in on cell populations and conduct more in-depth studies on them, revealing subtle changes unique to each cell, greatly advancing the field of genomics. It enables the fine distinction of different cell types, making it possible to study molecular mechanisms at the single-cell level. With the continuous development of next-generation sequencing technology, the first truly single-cell transcriptome sequencing technology (scRNA-seq) was developed in 2009 (9). Subsequently, with the benefits of more optimized cell sorting technology and nucleic acid amplification technology, single-cell nuclear RNA sequencing (10) technologies have developed rapidly.

As proteins directly mediate cell functions, it is important to understand their heterogeneity between cells. Traditional fluorescence-activated flow cytometry (FACS) uses up to 10 markers for single cells to avoid spectral overlap, while cytometry by time-of-flight (CyTOF) uses as many as 40 stable metal isotopes to label the protein of interest (11). In addition, the use of time-of-flight detectors to quantify the signal overcomes the limitations of single-cell proteomics analysis due to the lack of starting materials and the inability to directly amplify proteins, making it possible to analyze cell proteomics in the millions. Over the past few years, the single-cell transcriptomics has allowed researchers to delineate vascular cells at an unprecedented resolution. In this review, we will focus on how these collective studies have significantly advanced our understanding of vascular cell subtypes and functional characteristics in humans, mice, and monkeys.

## Subtypes of Immune Cells in Vasculature

Cardiovascular diseases are often accompanied by the infiltration of diverse immune cells (3, 12), especially macrophages, monocytes, B cells, T cells, dendritic cells (DCs), and natural killer cells (NK). The arrival of single-cell transcriptomics is now providing unprecedented opportunities to discover new immune cell subpopulations. Eleven immune cell subpopulations are identified in the aorta of atherosclerotic mice (13); however, only five or less subtypes are identified in healthy mice (14, 15), suggesting a high degree of immune cell heterogeneity in the diseased vasculature. Different species, tissue, disease, gender, genetic background, and exposure conditions will lead to different subtypes of immune cells (Table 1).

**Table 1:** Recent reports identified subtypes of immune cells in vasculature.

**Species**	**Gender and genotype**	**Tissue**	**Exposure**	**Method**	**Immunocyte subtype**		**References**
					**Total clusters**	**Marker (number of clusters)**	
Mus musculus	Female ApoE^−/−^	Aorta CD45^+^	WD 12 weeks, CHD	scRNA-seq, CyTOF, FACS	11	CD3e^+^ T(5), CD19^+^ B(2), CD11b^+^ myeloid(3), Klrb1c^+^ NK(1)	(13)
			CHD		5	CD3e^+^ T(1), CD19^+^ B(1), CD68^+^/H2ab1^+^ Macro(2), H2ab1^+^ Mono(1)	(13)
	Female WT	Aorta	CHD	scRNA-seq	3	Btg1^+^/H2ab1^+^ Macro(1), Lyz2^+^/C1qa^+^ Mono(2)	(14)
	WT	Aorta		scRNA-seq	9	B(2), T(4), myeloid (3)	(15)
	Male *Ldlr*^−/−^	Aorta CD45^+^	WD 28 weeks	scRNA-seq, FACS	11	CD64^+^/CD206^+^/CD11c^+^/Lyve1^+^ Macro(8), CD3e/d/g^+^/CD8^+^/Foxp3^+^T(1), Flt3^+^/Zbtb46^+^/CD103^+^ DC(2)	(16)
	Male WT, ApoE^−/−^	Aortic adventitia	CHD	scRNA-seq	12	CD14^+^/Cebpd^+^ Mono-Macro(4), CD79a^+^/CD19^+^ B(3), CD3d^+^ T(3), Il1rl1^+^/Gata3^+^ ILCs(1), Gzma^+^/Gzmb^+^/Klrb1c^+^ NK(2)	(17)
	Male *Ldlr*^−/−^	Aorta CD45^+^	HFD 11 weeks, CHD	scRNA-seq	14	Adgre1^+^/CD14^+^/Cxcl2^+^/Csf1r^+^/Trem2^+^ Macro(3), Ly6c2^+^/F10^+^/Lilrb4a^+^ Mono(1), CD79a^+^/Mzb1^+^ B(1), CD8b1^+^/Lck^+^/Cxcr6^+^/CD3d^+^ T(4), DC(2), Gzma^+^/Klrb1c^+^ NK(1), Furin^+^/Il1rl1^+^ Mast(1), Ngp^+^/Camp^+^ Granu(1)	(18)
	Male *Ldlr*^−/−^	Aorta CD45^+^	HFD 20 weeks	scRNA-seq	9	Adgre1^+^/CD9^+^/Pf4^+^/Il1b1^+^/Trem2^+^ Macro(3), CD79a^+^/Mzb1^+^ B(1), Cxcr6^+^/CD3g^+^ T(2), CD209^+^ DC(1), Gzma^+^/Klrb1c^+^ NK(1), Ly6d^+^ Mix T/B(1)	(18)
	ApoE^−/−^	Abdominal aorta	WD 2 weeks + Ang II 4 weeks	scRNA-seq	5	Adgre1^+^/CD11b^+^/CD68^+^ Macro(1), CD19^+^ B(1), Itgax^+^ NK(1), CD3e^+^ T(1), Ly6G/C^+^ Neutr(1)	(19)
	ApoE^−/−^	Aorta CD45^+^	HFD 12 weeks, CHD	CyTOF	13	CD68^+^/CD11b^+^/CD64^+^/Ly6C^−^ Macro(1), CD11b^+^/Ly6C^+^/CCR2^+^ Mono(1), CD11b^+^/Ly6G/C^+^ Neutr(1), CD11b^+^/SiglecF^+^ Eosin(1), CD11b^+^/CD11c^+^/MHCII^hi^/CD172a^+^ DC(3), CD19^+^ B(1), NK1.1^+^ NK(1), TCRß^+^/CD3^+^/CD4^+^/ CD8^+^/TCRγδ^+^ T(3), Lin^−^/CD90.2^+^/IL7Ra^+^ ILCs(1)	(20)
	Rank^Cre^Rosa26^eYFP^	Aortic adventitia CD45^+^		scRNA-seq	5	Adgre1^+^/CD11b^+^ Macro(3), Cd79b^+^/Cd19^+^/Ms4a1^+^/Ighd^+^ B(1), Trbc2^+^/Thy1^+^/Cd3g^+^/Cd3d^+^/Nkg7^+^ T/NK(1)	(21)
	Male 10-week-old C57BL/6J	Infrarenal abdominal aorta	Elastase-induced AAA at 7, 14, and 28 days	scRNA-seq	8	Mono/Macro(5), B(1), T(1), DC(1)	(22)
	Male 12-week-old C57BL/6J	Infrarenal abdominal aorta	CaCl_2_-induced AAA	scRNA-seq		DC(1), Neutr(1), NK(1), T/NK(1), B(1), Macro(3)	(23)
	Lyn^−/−^	Blood CD45^+^		CyTOF	20	DC(2), Neutr(1), CD90^+^/CD11c^+^/B220^+^ NK(3), T(5), B(4), CD115^+^ Mono(4), other CD45^+^ (1)	(24)
Homo sapiens	Male and female	Carotid artery plaques CD45^+^, blood CD45^+^		CyTOF, CITE-seq, scRNA-seq	15	DC(2), Neutr(1), NK(1), T(7), B(1), Cd14^+^ Mono(1), Macro(2)	(25)
		IPAH lung, healthy lung		Computational FACS	21	T(4), B(1), Neutr(1), Mast(1), Baso(1), Hla^+^/Cd1a^+^ Macro(4), Mono(3), CD11c^+^/CD209^+^ DC(6)	(26)
		IPAH lung, normal lung		scRNA-seq	7	T(1), B(1), Mast(1), Macro(1), DC(1), Club/Goblet/Basal(1), NK(1)	(27)
		ATAA aorta, healthy aorta		scRNA-seq	23	T(11), B(1), Mast(1), Macro(8), NAMPT^+^ Mono(1), FLT3^+^ DC(1)	(28)

*Different species, tissue, disease, gender, genetic background, and exposure conditions will lead to different subtypes of immune cells. CITE-seq, cellular indexing of transcriptomes and epitopes by sequencing; CyTOF, cytometry by time-of-flight; FACS, fluorescence-activated flow cytometry; IPAH, idiopathic pulmonary hypertension; WT, wild type; WD, Western diet; CHD, chow diet; HFD, high-fat diet; ILCs, innate lymphoid cells; Mast, mast cells; AAA, abdominal aortic aneurysm; ATAA, ascending thoracic aortic aneurysm; Macro, macrophages; Mono, monocytes; Neutr, neutrophils; Baso, basophils; Eosin, eosinophils*.

In 2018, the immune composition of human atherosclerotic plaques had been inferred from bulk RNA-seq data and CyTOF using a genetic deconvolution algorithm (13), emphasizing the diversity of immune cells in human plaques and suggesting the composition of immune cells is a potential driver of adverse clinical events. Two years later, Fernandez et al. (25) conducted single-cell proteomics and transcriptomics analyses on human carotid artery plaques to reconfirm and reveal the characteristics of innate and adaptive immune cells in plaques related to cerebrovascular clinical events. In patients with adverse clinical events, plaques are significantly infiltrated by activated T cells and proatherosclerotic macrophages, while the IL-1 β signaling pathway is activated in asymptomatic patients, suggesting that immunotherapy must be adjusted for specific immune deficiency. γδT cells serve as a bridge between innate and adaptive immune responses (29), in IPAH lungs, the main type of CD45^+^ cells change from neutrophils to T cells, with increases in γδT cell subtypes. Additionally, diversely activated classic myeloid dendritic cells, non-classical plasma cell-like dendritic cells, mast cells, and basophils are more abundant in IPAH lungs (26), macrophages may not be as active or may display more heterogeneous properties (27). The identification of different subtypes and rare cell subpopulations is of great significance to cardiovascular diseases.

## Subtypes of Macrophages in Vasculature

Monocytes in blood, together with tissue-intrinsic macrophages and dendritic cells (DCs), are classified as mononuclear phagocytes (30), contributing to tissue homeostasis and anti-inflammation (31). Monocytes differentiate into either M1 or M2 macrophages, with M1 macrophages usually considered proinflammatory and M2 macrophages considered anti-inflammatory (32).

### Macrophages in Atherosclerosis

A large number of macrophages accumulate in the atherosclerotic vascular wall (33) through different mechanisms (17, 34). Certain pathways are only enriched in certain subpopulations of macrophages (13), suggesting that macrophages function differently in regulating the progression of atherosclerotic lesions depending on the gene expression. Macrophages are classified into inflammatory macrophages, resident-like (Res-like) macrophages, and TREM2^hi^ macrophages (18), which are found together in the advanced stages of the disease. Inflammatory macrophages highly express atherogenic genes, macrophage Lgals3 (17) and M1, and Mox macrophage-related genes, suggesting that the feedback inhibition pathway of inflammatory response is activated. Res-like macrophages highly expressed chemokines and M2 macrophage-related genes Folr2, CD206, and CBr2 (17), with low expression of inflammatory genes Tnf and Il1b, suggesting a considerable anti-atherosclerotic phenotype. However, Res-like macrophages in the diseased adventitial aorta are up-regulated in cell-to-cell adhesion and immune cell migration pathways, which may participate in the response to hyperlipidemia and thus attract immune cells, suggesting their potential role in triggering adventitial inflammation (17). TREM2^hi^ macrophages have a unique genetic profile with high expression of CD9 (functions in cell differentiation, adhesion, and signal transduction), Spp1 (encoding osteopontin), and Hvcn1 (facilitates acute production of reactive oxygen species), suggesting a specific role in lipid metabolism, catabolic metabolism, and lesion calcification. As the disease progresses, the relative increase of total macrophages in CD45^+^ leukocytes is mainly due to the increase of Res-like macrophages and inflammatory macrophages, suggesting that changes in macrophage frequency may occur in advanced atherosclerosis. However, it remains unclear whether absolute changes in the abundance or localization of these macrophage subtypes or their effectors will promote the progression of lesions to advanced plaque phenotypes. The verification of RNA quantification or protein localization of these cell subtypes will greatly improve biological insight, which is lacking in some of the latest reports.

When we try to understand the pathogenesis of atherosclerosis and explore new therapeutic targets, the formation of foamy cells and its role in atherosclerosis are important topics (35). Using fluorescent lipid probes combined with FACS and scRNA-seq technology reveals that macrophage subtypes exhibited unique transcriptome profiles according to their lipid content (16). However, further studies are required to understand the exact origin and characteristics of plaque macrophages. For instance, scRNA-seq analysis of plaque macrophages isolated from early or advanced plaques will help to dissect macrophages subsets in lesions and understand the phenotypic changes of macrophages during plaque progression. Furthermore, detecting the molecular dynamics of cell fate determination help researchers understand and correct abnormal macrophages behavior during atherosclerosis. Lin et al. (36) used scRNA-seq combined with lineage tracing technology in aortic plaques found that monocyte-derived macrophages in lesion progression and regression stages were divided into 11 subpopulations, of which the four most differentially expressed subpopulations were Folr2^hi^ macrophages, chemokine^hi^ macrophages, TREM2^hi^ macrophages, and NMES1^hi^ macrophages, confirming the previous findings of inflammatory macrophages, Res-like macrophages, and TREM2^hi^ macrophages all derived from monocytes and representing the general inflammatory state of atherosclerosis (18). Many macrophage populations shared common characteristics between progression and regression states, which highlights that treatment based on progressing plaque may have unexpected adverse consequences on the regression process. Therefore, it's worth exploring which macrophage subtypes and mechanisms play a key role in plaque regression, the characteristic rapid enrichment of M2 macrophages in mouse atherosclerosis regression models is sufficient for the resolution of inflammation, it depends on STAT6-dependent signaling in the newly recruited monocytes suggest that local factors in the regressing plaque stimulate this signaling enrichment pathway (37). Therapeutic strategies that promote M2 macrophage aggregation in plaque are potential methods to promote plaque regression.

### Macrophages in Aortic Aneurysm

During the progression of AA, a significant change of adventitia is the infiltration of macrophages into lesion (3). Macrophages within vascular wall display diverse functions, including amplifying local inflammatory response by secreting pro-inflammatory cytokines, chemokines, proteases, and reactive oxygen species (3, 38, 39).

There are several AA mouse models that recapitulate important features of human AA, in a CaCl_2_ model, inflammatory macrophages cluster is responsible for the accumulation of macrophages at an early stage in abdominal aortic aneurysm (AAA) development (23); however, the function of a proliferative cluster in AAA remains to be proven. In the infrarenal abdominal aortas (IAAs) from C57BL/6J mice who undergo elastase-induced AAA, five monocyte/macrophage clusters are identified (22). Notably, the authors determine the expression of M1 and M2 macrophage markers in all of the five monocyte and macrophage clusters. Thus, a panel of traditional markers for M1 or M2 macrophages are not sufficiently specific to functionally characterize the cells that express them. Inflammatory macrophages are responsible for the accumulation of macrophages at an early stage in AAA development.

A huge heterogeneous distribution of macrophage clusters is identified in the ascending thoracic aortic aneurysm (ATAA) of patients (28), playing roles in tissue remodeling, glucose metabolism, anti-inflammation, and phagocytosis. Furthermore, the dynamic mechanism of bridging persistent inflammation and focal ECM erosion has been revealed, and netrin-1 (Ntn1) is identified as an instrumental signal to maintain this destructive response (19). In this study, the immune cells account for no more than 10% of the total cells in the angiotensin II-induced AAA model. However, in Zhang's study (22), immune cells accounted for more than 60% of total cells isolated from elastase-treated IAAs, this variation is more likely the result of the intrinsic differences in the pathophysiological aspects of the two models, besides, it is important to note that although scRNA-seq data is valuable for analyzing gene expression changes in different populations, it is not the best way to estimate the proportion of cell populations, and more accurate methods, such as FACS, are needed to confirm or deny the actual cell composition. Detailed information about subtypes and functional characteristics of macrophages in vasculature are presented in Supplementary Table 1.

## Subtypes of Monocytes in Vasculature

Monocytes are a heterogeneous group of cells, divided into classical monocytes, non-classical monocytes, and intermediate monocytes. The functions of classical and non-classical monocytes have been clearly defined. The former has pro-inflammatory effects in atherosclerosis (40), the latter plays a role in eliminating inflammation (37, 41), while the functions of intermediate monocytes are mixed (42).

Mouse monocytes are classified into Ly6C^hi^ classical monocytes (cMo), Ly6C^lo^ non-classical circulating monocytes (pMo), and intermediate monocytes (intMo) (43–45). pMo has been shown to play a protective role in atherosclerosis (44) and Lck/yes novel tyrosine kinase (LYN) might serve as a negative regulator of pMo homeostasis (24). Monocytes in human peripheral blood account for about 10% of all circulating immune cells and are classified into classical CD14^++^ CD16^−^ monocytes, non-classical CD14^+^ CD16^+^ monocytes, and intermediate CD14^++^ CD16^+^ monocytes. Recent studies have proved that human monocytes are quite heterogeneous (46), intermediate monocytes are divided into two subpopulations, one of which is cytotoxic (47), single-cell transcriptomics can better define monocyte subpopulations (48). Changes in monocyte profiles are also a feature of human atherosclerotic plaques (13). In the blood of patients with severe coronary heart disease, the frequency of Slan^+^ non-classical monocyte subtypes is significantly increased and shows enhanced exocytosis capacity, indicating its role in protecting atherosclerosis, but also suggesting that Slan protein expression may be an important biomarker for the progression of coronary heart disease (46). A summary of recent reports identified subtypes and functional characteristics of mononuclear phagocytes in vasculature are presented in Supplementary Table 1. The transcriptomic profile of human coronary mononuclear phagocytes remains to be deeply elaborated. In fact, the preparation of mononuclear phagocytes for scRNA-seq analyses from human coronary is still highly challenging. However, it is very important to demonstrate whether the transcriptomic profiles of human mononuclear phagocytes are comparable with those of mice, which will provide new immunological insights into the innate immune responses in atherosclerosis.

## Subtypes of T Cells in Vasculature

More and more evidence showed that the activation of adaptive immune response is related to the occurrence and development of atherosclerosis. Cells expressing MHC II in atherosclerotic plaques at various stages of the disease form the basis of adaptive immune response of atherosclerosis (49). Treg is a specific subtype of T cells, which has been proved to play a leading role in maintaining tolerance and immune homeostasis (50). A team found that the spleen Treg of ApoE^−/−^ mice showed a unique non-inhibitory IFNγ^+^ Th1/Treg phenotype, indicating that with the development of atherosclerosis, the immunosuppressive phenotype of Treg cells disappeared, revealing the damage of phenotype transformation of Treg cells in atherosclerosis (51). Among the CD4^+^ T cell subpopulations of aorta, one subpopulation is transcriptionally similar to memory T cells, and shows west diet (WD)-induced cell cycle gene enrichment, suggesting a tissue-specific proliferative response (13).

Innate lymphoid cells (ILCs) are located in the mucosal barrier and play an important role in host defense and tissue homeostasis. ILCs are composed of cytotoxic and non-cytotoxic cells and are similar to T lymphocytes in terms of cytotoxicity and cytokine production (52). In the aortic adventitia of ApoE^−/−^ mice, the genes related to IL1 response and oxidant detoxification are upregulated in ILCs subpopulation, and NF-κB and nucleotide-binding oligomer domain like receptor (NOD) signaling pathways related to innate immunity are upregulated, which further reflect the function of T cells and represent a new immunological pathway (52), indicating that ILCs play an important role in regulating the development of lesions (17).

Using single-cell proteomics and transcriptomics to analyze the heterogeneity between plaques and blood T cells in patients with acute ischemic events reveal the unique differences and highly specialized functions of T cells, and highlight new immune disorders in plaques associated with clinical complications. For example, Fernandez et al. (25) found that T cells in carotid plaque of asymptomatic patients and patients with symptoms were divided into four subpopulations. The plaque of symptomatic patients was rich in CD4^+^ T cells. Pathways of T cell migration (RhoGTPase), activation (PDGFR-β), and differentiation (Wnt, IL-2) are upregulated. T cells were not only prominent in plaques but also had stronger activation, differentiation, and exhaustion capacity than those in blood. In the plaque of asymptomatic patients, most T cells were activated, IL-1 β signaling pathway and IL-1, IL-6 signaling pathways were upregulated, which stabilized cytokine transcripts and promoted T cell survival. However, IL-1 and IL-6 signaling pathways of T cells in the plaque of symptomatic patients with CD4^+^ abundance were inhibited, which indicated that drugs targeting the above pathways may be ineffective after acute ischemia, and new immunotherapy must be precisely customized for immune cell subpopulations (25).

## Subtypes of B Cells in Vasculature

Atherosclerosis is a chronic inflammatory process. Inflammation is related to the accumulation of low-density lipoprotein (LDL). The B1 cell-derived IgM antibody against the specific oxidized epitope of LDL has anti-inflammatory and anti-atherosclerotic effects. Bone marrow B1a cells from ApoE^−/−^ mice express specific IgM antibodies compared with peritoneal B1a cells, and some of them shared the same site sequence, indicating that B1a migrated between bone marrow and abdominal cavity. Further studies have found that mature abdominal B1a cells migrate to bone marrow in a CXCR4 dependent manner, while the expression of CXCR4 on B1 cells is higher in people with smaller coronary plaque areas, which reveals a potential target immunomodulatory method that can limit atherosclerosis (53). Different B cell subpopulations may play different roles in atherosclerosis. B cells in plaque aorta are divided into three subpopulations. The CD11b^+^CD5^+^ B1 subpopulation shows the function of antigen presentation, antibody production and cell adhesion, which is consistent with the protective effect of B1 cells. Two B220^+^CD23^+^ B2 cell subpopulations overexpress apoptotic genes, pro-atherosclerotic factor IFN-γ, and recombinant macrophage granulocyte colony stimulating factor (GM-CSF), suggesting that B2 cell subpopulations can promote atherosclerosis.

Taken together, more and more studies use single-cell transcriptomics to elucidate the role of various immune cell populations after cardiovascular disease, which improves our understanding of the changes of microenvironment in disease progression and regression. Future research will benefit from a more in-depth study of the specific functions of these cell types and transition states, especially at different stages after disease. At present, in disease progression and regression, transcriptome research and subsequent verification of intercellular communication between immune cells and non-immune cells have not been fully studied. To fill these gaps in our knowledge is critical to improving our understanding of the cellular responses involved in the different stages of diseases.

## Subtypes of Endothelial Cells in Vasculature

Endothelial cells (ECs) line the surface of blood vessel lumen and have the functions of actively regulating blood flow, maintaining blood fluidity, controlling the transfer of solutes, and macromolecules between blood and tissues, and regulating the recruitment and activation of circulating immune cells. Dysfunction of ECs lead to various vascular diseases. The heterogeneity of ECs has been recognized by more and more experts in vascular biology. Unbiased analysis of single-cell genomics helps to reveal the role of ECs heterogeneity in vascular physiology and pathology, a summary of recent reports identified subtypes and functional characteristics of ECs in vasculature is presented in Table 2.

**Table 2:** Recent reports identified subtypes and functional characteristics of endothelial cells in vasculature.

**Species**	**Gender and genotype**	**Tissue**	**Exposure**	**Method**	**ECs subtypes**				**References**
					**Number of clusters**	**Name**	**Gene expression**	**Function**	
Mus musculus	Female WT	Aorta	CHD	scRNA-seq	3	EC1	CDh5, Vcam1, Clu, Gkn3, Eln	ECM integrin	(14)
						EC2	CDh5, Cd36, Fabp4, Lpl, Pparg, Flt1	Lipid transport angiogenesis	
						EC3	CDh5, Lyve1	Lymphatic	
			WD	scRNA-seq		ECs	Myl9, Tagln, Acta2	EndMT	
	Male ApoE^−/−^	Aortic adventitia	CHD	scRNA-seq	1	Adv-ECs		Inflammatory	(17)
	Male ApoE^−/−^	Aorta	WD 16 weeks	scRNA-seq		ECs	Icam1, Vcam1, Il6, Ccrl2, S1pr3, Darc, Gpr153	Inflammatory	(54)
	WT	Lung	LPS	scRNA-seq		ECs	Icam1, Vcam1, Sele, Ccrl2, Cxcr7, Gpr111	Inflammatory	
	Tie2-Cre; Lmna^G609G/G609G^	Lung		scRNA-seq	1	ECs	Icam1, p21^Cip1/Waf1^	Aging	(55)
	Young WT	Aorta		scRNA-seq		ECs	Atf3, Fox, Jun, Egr1, Klf4, Klf2	Regenerative	(56)
	Female WT	Descending aorta		scRNA-seq	3	EC1	CD34, Pecam1, Jchain, Rgs5, Egfl7, Sox17, Cd36, Fabp4, Cldn5	Mature	(57)
						EC2	CD34, Pecam1, Pdgfra, Il33, Serpinf1, Lum, Mfap5, Smoc2, Dcn, Lum, Gsn	Mesenchymal	
						EC3	CD34, Pecam1, H2-Ab1, Hmgb2, H2-Eb1, H2-Aa, Il1b, Aif1	Terminal differentiation, inflammatory	
	WT	E11.5 AGM		scRNA-seq	2	AECs VECs	VEGFA, FZD4, FZD7, FZD10, Dll4, NOTCH1	Protective	(58)
	WT	E14.5 endocardium and coronary endothelium		scRNA-seq	2	Fabp4^+^	Pecam, Cdh5, Fabp4, Nfatc1		
						Npr3^+^	Pecam, Cdh5, Nfatc1, Npr3	Endocardium specific	(59)
	ApjCreER, Rosa^mTmG^	E12.5 GFP^+^	Tamoxifen	scRNA-seq		SVc	Slc45a4, Dll4, EFNB2, Cx40, Mecom, Igfbp3	Arterialization	(60)
Macaca fascicularis	Aging	Aorta and coronary arteries		scRNA-seq		ECs	Colec10, Zbtb16, Tcf3, Myc, Thbs4, Pik3r1, Sorbs1, Txnip	Aging	(61)
Homo sapiens		CS13 AGM		scRNA-seq	3	ECs	Aplnr, Nrp2, Nt5e	Classical	(62)
						AECs	Gja5, Gja4, Hey2, Cxcr4	ECM, vascular development	
						HECs	Runx1, Snhg16, Rpsap58, Ldhb	Hematopoietic	
	Male and female	ATAA aorta, healthy aorta		scRNA-seq	2	EC1	Vwf, Pecam1, Pecam17, Ifi27, Emp1	High cell-cell junction	(28)
						EC2	Vwf, Pecam1, Pecam17, Ifi27, Slc9e3r2	Higher cell-cell junction	
		Pulmonary artery		Comparative single-cell transcriptomics		ECs	Unique P2 receptor Ca^2+^ signaling body features	Pulmonary hypertension	(63)
	Male and female	IPAH lung, normal lung		scRNA-seq	2	ECs	Vwf, Pecam	Classical	(27)
						Lymphatic ECs	Vwf, Pecam, Cav1	Lymphatic	

*A summary of recent reports identified subtypes and functional characteristics of endothelial cells in vasculature. E11.5, embryonic stage 11.5; AGM, aorta-gonad-middle kidney; CS, carnegie stage; ECs, endothelial cells; AECs, arterial endothelial cells; VECs, venous endothelial cells; HECs, hematopoietic endothelial cells; WT, wild type; WD, Western diet; CHD, chow diet; HFD, high-fat diet; ATAA, ascending thoracic aortic aneurysm; IPAH, idiopathic pulmonary artery hypertension*.

### Endothelial Cells in Coronary Artery Development

Further understanding of the fate transition mechanism of ECs through single-cell transcriptomics combined with lineage tracing technology provides potential therapeutic applications. Zhang et al. (59) resolved the controversial issue of whether the coronary endothelial cell (EC) originated from the ventricular endocardium or the sinus vein. They identified a new type of endocardial gene, natriuretic peptide receptor 3 (Npr3), which was specifically expressed in the endocardium. Resolving this controversy is crucial for understanding the molecular mechanism of coronary artery development.

Cardiac sinus vein ECs undergo a gradual transformation from vein endothelial cells (VECs) to arterial endothelial cells (AECs), which form pre-arterial cells and finally develop into coronary arteries. Previously, it was believed that the development of coronary artery was only related to the aortic trunk, which could provide embryonic blood flow (64). However, scRNA-seq provides a unique insight into the phenotypic transformation of VECs to pre-arterial cells before coronary artery perfusion and determines transcription factors COUP-tf2 as the inhibitor (60), future experiments should examine whether this high-resolution understanding of coronary artery differentiation during cardiac angiogenesis could promote the development of regenerative therapy.

Besides, study of AEC's fate transitions and inhibitory signals may also improve understanding of coronary development and promote regenerative medicine. The efficient induction of functional AECs is one of the challenges of regenerative therapy. scRNA-seq identifies molecular pathways that increase the efficiency of generating functional AECs from human pluripotent stem cells, helping to develop a five-factor induction therapy (Fgf2, VEGFA, SB431542, RESV, and L690) to promote AEC differentiation (58), the AECs are able to significantly enhance survival rates in a mouse myocardial infarction model, but the mechanism underlying this improvement is unclear.

### Endothelial Cells in Aorta and Lung

The unbiased transcriptional analysis shows that ECs are divided into three subpopulations: EC 1 specifically expresses classical EC markers Vcam1; EC 2 specifically expresses lipid-handling genes including Fabp4 and the angiogenesis marker Flt1; and EC 3 expresses markers of lymphatic identity including Lyve1 (14), revealing specific functions in extracellular matrix production, lipid processing, and angiogenesis or lymphatic function. Compared with normal mice, ECs in diseased mice show significant changes in the expression of functionally relevant genes. For instance, during atherosclerosis, chemokine activity, CCR receptor activity and arachidonic acid-binding capacity of adventitial ECs are upregulated ^25^, two EC subpopulations in septic mouse lung endothelial cells upregulate inflammatory activation markers Icam1, Vcam1, Sele, and some GPCRs (54). Endothelial to mesenchymal transition (EndMT) in atherosclerosis is associated with upregulation of smooth muscle cell markers in ECs (14, 65), corroborating the EC's low expression of Myh11 found by Kaur et al. (54), suggesting that EndMT is the result of contractile transcriptional upregulation in all EC subpopulations.

ECs displayed the most differentially expressed genes between idiopathic pulmonary artery hypertension (IPAH) and normal lungs (27), comparative single-cell transcriptomics of human ECs from 11 different tissues, and organs reveals the unique P2 receptor Ca2^+^ signaling body signature of human pulmonary artery endothelial cells, which is susceptible to genetic perturbations that affect vascular integrity, inflammatory and thrombotic responses, as well as survival and DNA repair, leading to pulmonary hypertension (63). In idiopathic pulmonary fibrosis (IPF), GDF15 is identified as a novel cell-specific marker of epithelial injury and a novel biomarker of IPF severity (66). GDF15 levels have also been associated with cellular senescence, it is necessary to conduct more studies on the larger cohort to comprehensively assess the relationship between aging and GDF15 expression. The contribution of GDF15 to age-related phenotypes and how to distinguish cellular senescence from cellular stress is unclear.

## Subtypes of Smooth Muscle Cells in Vasculature

Vascular smooth muscle cells (VSMCs) are located in the vessel wall. As the largest vascular cell population (14), VSMCs provide mechanical support to vessels and regulate vascular tension to control blood flow and blood pressure. In the past 30 years, the plasticity of VSMCs has been extensively studied *in vitro* (67, 68). From these studies, the concept of phenotypic switching of VSMCs has been proposed—the switching between differentiated, contracted phenotype and dedifferentiated, secreted phenotype. The former expresses typical contractile proteins αSMA, MYH11, SM22α, and CNN1. When stimulated by injury or inflammation, VSMCs downregulate the expression of contractile genes, increase extracellular matrix (ECM) synthesis, and enhance secretion, migration, and proliferation (69), a motility-associated VTN^high^ VSMCs has been identified as an intermediate switching type (15). Loss of VSMC characteristic is a key factor in vascular diseases such as atherosclerosis, aortic aneurysm, pulmonary hypertension, and restenosis (70). Thus, searching for factors critical for VSMC gene expression may eventually lead to effective therapies. More and more studies have demonstrated that VSMCs are highly heterogeneous. A summary of recent reports identifying subtypes and functional characteristics of VSMCs in vasculature are presented in Table 3.

**Table 3:** Recent reports identified subtypes and functional characteristics of smooth muscle cells in vasculature.

**Species**	**Gender and genotype**	**Tissue**	**Exposure**	**Method**	**SMCs subtypes**				**References**
					**Number of clusters**	**Name**	**Gene expression**	**Function**	
Mus musculus	Female WT	Aorta	CHD	scRNA-seq	1	VSMCs	Myh11, Tpm2, Myl9, Tagln, Acta2	Classic	(14)
	Male WT	Aorta		scRNA-seq	7	Sca1^+^	Mgp, Col8a1, Spp1, Pak3, Igf1, Igfbp5, Sca1	Stimulated	(71)
	Male ApoE^−/−^Myh11CreER^t2^; Confetti^f/f^	Aorta	Tamoxifen + WD 14~18 weeks	scRNA-seq	9	Ly6a/Sca1^+^	Myh11^low^, sca1, Acta2^low^, Tagln^low^, Cnn1^low^, Mgp, Col8a1, Spp1, Pak3, Igf1, Igfbp5, PI3K, GTPases, Tgf-beta	Stimulated	
						Ly6a/Sca1^−^	Myh11^low^, Acta2^low^, Tagln^low^, Cnn1 ^low^, Sox9, Ibsp, Chad	Calcified	
	Male *ApoE*^−/−^*Tg^*Myh*11−*CreERT*2^ ROSA^*tdT*/+^*	Aortic root and ascending aorta	Tamoxifen + HFD 0/8/16 weeks	scRNA-seq	3	Modulated VSMCs	Tagln^low^, Cnn1^low^, Lgals3^low^, Fn1, Tnfrsf11b, Col1α1, Lum, Dcn, Bgn, Tcf21	Fibrogenic	(72)
						Contracted VSMCs	2 subtypes, Tagln^high^, Cnn1^high^, Lgals3^high^		
	Male ROSA26^ZsGreen1/+^; Ldlr^−/−^; Myh11-CreER^T2^	Aorta	Tamoxifen + WD 0/8/16/26 weeks	scRNA-seq	6	SMC	Acta2, Myh11	Contractile	(73)
						SEM	Ly6a, Vcam1, Ly6c1, Lgals3	MSC-like	
						FC	Fn1, Col1a1, Col1a2, Col3a1	Extracellular matrix	
						Macrophage-like1/2/3			
	Male and female WT	Ascending aorta	Ang II 1 weeks	scRNA-seq	6	Sting^hi^	2 subtypes, Acta2, Myh11, Mylk, Casp9, Htra2, Tnfsf15, Mkl, Stat1, Zbp1, Gsdmd, Mmp13, Cxcl13, Cxcr3, Cxcr2	Degraded	(74)
	Male 10-week-old C57BL/6J	Infrarenal abdominal aorta	Elastase-induced AAA 7/14/28 days	scRNA-seq	4	SMC_1	Myh11, Acta2, Tagln, Myl9	Quiescent, contractile	(22)
						SMC_2	Myh11, Acta2, Tagln, Myl9, Fos, Jun, Klf2, Atf3	Proliferative, contractile	
						SMC_3	Myh11, Acta2, Tagln, Ifrd1, Dusp1, Mt1, Mt2	Dedifferentiated	
						SMC_4	Myh11, Acta2, Tagln, Ifrd1, Nrip2, Klf4, Notch3, Cd68	Inflammatory like	
	Male 12-week-old C57BL/6J	Infrarenal abdominal aorta	CaCl_2_-induced AAA	scRNA-seq	2	SMC-1	Myh11, Acta2, Tagln	Contractile	(23)
						SMC-2	Acta2, Aqp1	Synthetic	
	Male and female Fbn1^C1041G/+^	Aortic root/ascending aorta	4 weeks/24 weeks	scRNA-seq	2	SMCs	Acta2, Myl9, Myh11, Tpm2		(75)
						Modulated VSMCs	Acta2, Myl9, Myh11, Tpm2, Fn1, Mgp, Nupr1, Eln, Mmp2, Tnfrsf11b, Tgfb1	Fibrogenic	
Homo sapiens	Male	Marfan syndrome	25 years	scRNA-seq	2	Modulated VSMCs	Col1a1, Ctgf, Serpine1, Tgfb1	Fibrogenic	(75)
	Male and female	Carotid plaque			2	H2AFZ^High^	Cald1, Acta2, Mylk, Aebp1	Contracted	(76)
						H2AFZ^Low^	Smarca4	Secretory	
		ATAA aorta, healthy aorta		scRNA-seq	8	Stressed SMC	Actc1, Myl9, Acta2, Fos, Atf3, Jun, Hspb8	Stress response	(28)
						Contractile SMC	Actc1, Myl9, Acta2	Contraction related	
						Proliferating SMC1	Gas6, Igfbp2, Mgp, Fth1, Myh10	Higher proliferation	
						Proliferating SMC2	Cald1, Myh11, Map1b, Sparc, Fgf1	Higher proliferation	
						Fibromyocytes	Acta2, Myl9,Col1a2, Col8a1	Cell-ECM junction	
						Inflammatory1	Cxcr4, Btg1, Acap1, Dusp2, Ccl4, Rel, Srgn	T lymphocyte like	
						Inflammatory2	C1qa, C1qb, Cd74, Fcer1g, Aif1, Maf	Macrophage like	
						Inflammatory3	Ifit1, Isg15, Ifi6	Interferon induced	

*A summary of recent reports identified subtypes and functional characteristics of VSMCs in vasculature. WT, wild type; WD, Western diet; CHD, chow diet; HFD, high-fat diet; SEM, SMC-derived intermediate; FC, fibrochondrocyte-like cells; MSC, mesenchymal stem cells; ATAA, ascending thoracic aortic aneurysm*.

### Smooth Muscle Cells in Atherosclerosis

VSMCs of healthy mice can be divided into seven subpopulations, including one subpopulation expressing rare Sca1. This subpopulation is upregulated in genes related to ECM synthesis (Mgp, Col8a1, and Spp1), migration (Pak3, Igf1, and Igfbp5), and growth factors activation, with downregulation of contractile phenotype, which is assumed to be MSC (77). However, SMC fate mapping and scRNA-seq of both mouse and human atherosclerotic plaques reveal SMC-derived intermediate cells, termed “SEM” cells, are more complex and could differentiate into macrophage-like and fibrochondrocyte-like cells (73), the modulated SMC, “fibromyocytes” identified by Wirka et al. (72) contain both the SEM cell and fibrochondrocyte-like cells, suggesting that SEM cells are not simply MSC like, but a unique transition state from SMC in atherosclerosis.

In contrast to stable coronary atherosclerotic plaques, unstable plaques are characterized by the presence of numerous necrotic lipid nuclei and microscopic fibrous caps that are highly susceptible to rupture. VSMCs are involved in the formation of the fibrous cap and the underlying necrotic core through phenotypic transformation (78). VSMC subpopulations in the aortic root of SMC-specific aryl hydrocarbon receptor (AHR)-knockout mice were divided into six subpopulations, including two FMC subpopulations. The AHR was predominantly expressed in the FMCs, which was linked to a molecular pathway downstream of Tcf21 (72). SMC-specific knockout mice with AHR had significantly larger plaque lesions and a shift from FMCs to a chondrogenic SMCs (79); however, the existence of AHR-specific SMCs in human plaque and the exact mechanisms by which regulate SMC transition is still being defined.

Aberrant epigenetic changes may be caused by changes in the activity of enzymes that promote the binding or dissociation of genes to covalent groups, alter the epigenome, and then regulate gene expression. Identification of new individual epigenetic modifications at the single-cell level creates conditions for disease prediction, diagnosis, and prognosis, as well as the development of new therapeutic strategies. Schiano et al. (76) identified the H2A.Z gene from human carotid plaque VSMCs, which was significantly enhanced in the ACTA2^High^ subpopulation, suggesting a relationship between H2A.Z gene expression and VSMCs phenotype. The VSMCs of H2AFZ^High^ were contractile and those of H2AFZ^Low^ were secretory, indicating that H2A.Z plays an essential role in maintaining the VSMCs phenotype.

### Smooth Muscle Cells in Aortic Aneurysm

Dynamic SMC phenotype modulation promotes extracellular matrix substrate modulation in aortic aneurysm (23). Two VSMC subpopulations in the aorta overexpress interferon gene-stimulating factor (STING) in aneurysm. STING is involved in the induction of multiple stress responses in VSMCs, including oxidative stress responses, DNA damage responses, inflammatory responses, and activation of cell death pathways that promote aortic degeneration and cause aneurysm (74). Comparison with atherosclerotic aortic data reveals similar patterns of SMC modulation but identifies an Marfan syndrome (MFS)-specific gene signature in the modulated SMCs. Collectively, scRNA-seq implicates TGF-β signaling as potential upstream drivers of SMC modulation (75). TGF-β pathway is closely related to the pathogenesis of aneurysm in elderly patients with atherosclerosis. After knockdown of Tgfbr2 in the aortic VSMCs of atherosclerotic mice, a subpopulation of VSMCs is transformed into mesenchymal stem cell-like cells, associated with the formation of osteoblasts (aggrecan), chondrocytes (osteopontin), adipocytes (adiponectin), and macrophages (CD68) at the site of aneurysm lesions (80).

VSMCs within and between different vascular regions are heterogeneous in morphology, growth characteristics and specific gene expression (71), thus the curved structure of the arch is disease susceptible. The identity of VSMCs is controlled by a highly coordinated regulatory network, in which environmental cues, signaling transduction kinases, transcription factors, and chromatin states coordinate to determine VSMC-specific gene expression program (69, 76, 81). Validation of factors as revealed by scRNA-Seq may help us complement the network of VSMC identity regulation, with the ultimate goal of providing a therapeutic strategy to manipulate VSMC fates in development and diseases.

### Smooth Muscle Cells in Pulmonary Hypertension

It is well-established that VSMCs play a vital role in the initiation and progression of pulmonary hypertension by modulating the contractility and proliferation of SMCs to remodel the pulmonary vasculature. By using single-cell transcriptomics, smooth muscle cells were found to express genes implicated in the regulation of cellular apoptosis and extracellular matrix organization in the patients of pulmonary hypertension (27), suggesting that these cells converted to a profibrotic phenotype.

## Discussion

In this review, we described not only macrophage but also other immune cell and mesenchymal cell subsets in three major cardiovascular diseases: atherosclerosis, aortic aneurysm, and pulmonary hypertension. Furthermore, we summarized these types of cells' subset-specific markers and functions as shown in Tables 1–3 and Supplementary Table 1. Through these literatures, we found that single-cell transcriptomics is not a technology, but a concept/insight/dimension. Just like the concept of “atom,” it provides a new perspective for us to rethink the basic composition of subjects. Profiling the complete vascular milieu using single-cell transcriptomics provides key insights for disease pathogenesis.

One of the challenges in single-cell experiments is the preparation of high-quality single-cell suspension for downstream analysis. To isolate high-quality cell suspension from vascular tissues, an enzyme cocktail is better than a single enzyme, for example, the combination of collagenase type II, collagenase type XI, soybean trypsin inhibitor, elastase lyophilized, hyaluronidase type I, and HEPES (28). Besides, tissue dissociation should be examined frequently under a microscope to evaluate the dissociation efficiency. Moreover, a well-designed single-cell experiment is sufficiently powered to obtain high-quality data. Different from ordinary bulk RNA seq, each cell is unique and the sample is not easy to obtain. Therefore, it is necessary to determine the optimal number of repeats. Generally speaking, the negative control or normal group needs at least two biological repeats, and the experimental group may need more than two repeats.

By simultaneously analyzing the transcriptional signature of major vascular cell types, we partition the relative contribution of the four vascular cell types to cardiovascular diseases (macrophages, monocytes, endothelial cells, and smooth muscle cells). The classification and proportion of cell subpopulations in vasculature vary with species, location, genotype, and disease, exhibiting unique gene expression characteristics in organ development, disease progression, and regression. Specific gene markers are expected to be the diagnostic criteria, therapeutic targets, or prognostic indicators of diseases. Therefore, treatment of vascular disease still has lots of potential to develop.

### Clinical Significance

In detection and diagnosis, the emergence of single-cell transcriptomics has brought us many new opportunities, especially *in vitro* diagnosis. The rise of *in vitro* diagnosis is mainly due to cancer and rare diseases, for which the diagnosis is complex. If the diagnosis is unclear, we cannot develop a stable treatment plan, as one scholar said: “you can't stop it if you can't see it.” Based on single-cell transcriptomics, we can map the development track of human embryos; Tang (82–84) did a lot of meaningful work in this area, depicting the picture of embryo development. The diagnostic significance of single-cell level is undoubtedly huge for cancer and rare diseases (85–87), because they have heterogeneity, and single-cell transcriptomics is a powerful weapon to reveal heterogeneity. This is an important supplement to the previously accumulated bulk data; after a period of time, bulk data will become a supplement to single-cell data.

In clinical practice, diagnosis is not enough; the next stage is the prevention and treatment of diseases. Prevention refers to the development of vaccines, and treatment refers to the development of drugs. One of the main challenges of healthcare is that patients respond differently to a treatment. At present, about 90% of the drugs on the market are ineffective for more than 50% of patients (88). More and more patient-specific cell heterogeneity is considered to be the underlying cause and driving force of differences in drug response between individuals. With the development of single-cell transcriptomics, drug development will be greatly carried out.

Although the logic of drugs and vaccines is different, they are inextricably linked in immunology. The role of single-cell immune repertoire in immunotherapy is obvious. High-throughput single-cell RNA and VDJ sequencing were used to rapidly identify neutralizing monoclonal antibodies (mAbs) against neocoronavirus from antigen-enriched B cells of convalescent patients (89), which can undoubtedly provide support for the development of monoclonal antibody drugs.

**Figure 1:**
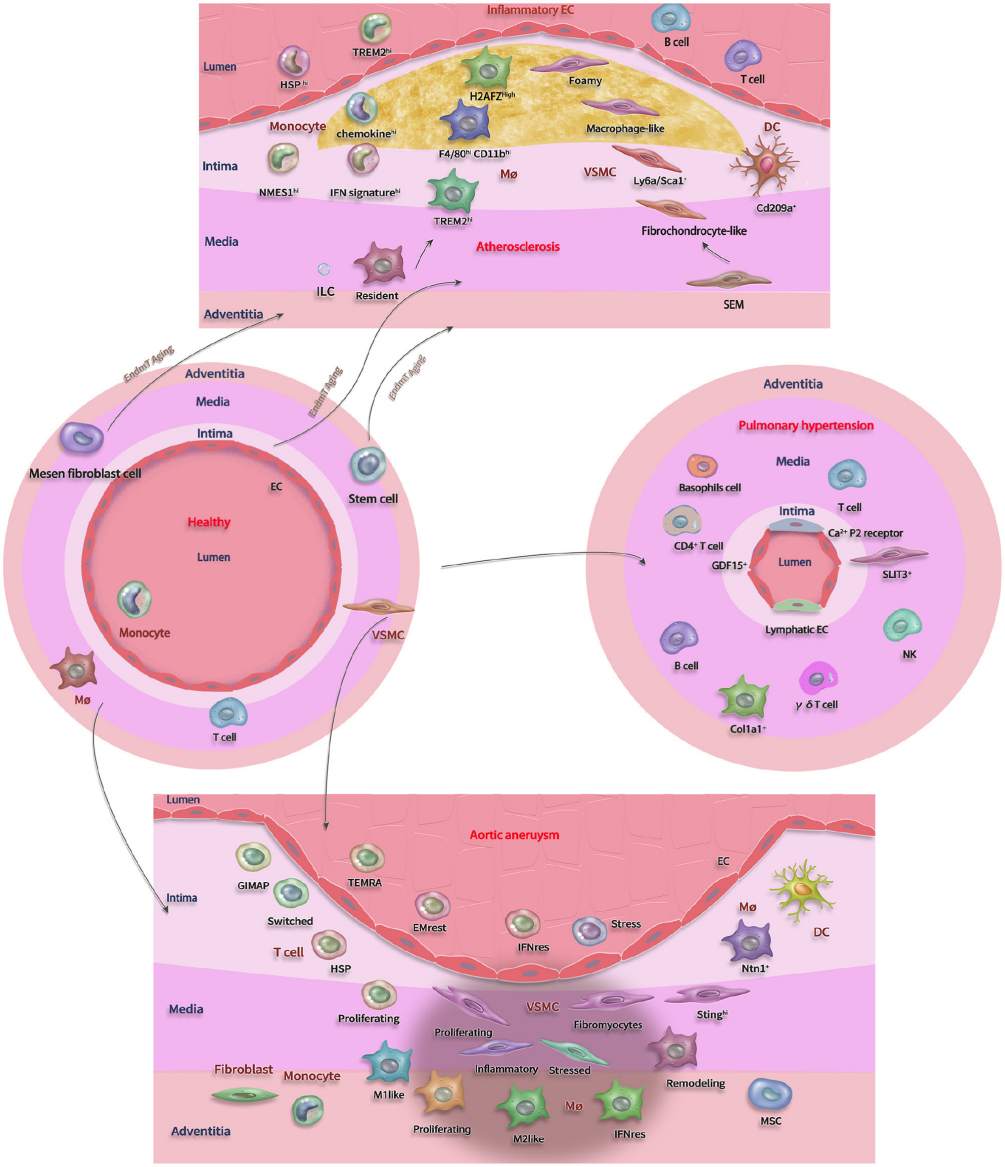
A schematic overview of vascular cell types and their heterogeneous subtypes. The different layers of the vasculature (adventitia, media, and intima) and the different condition (healthy, atherosclerosis, aortic aneurysm, and pulmonary hypertension) with involved cell types. The graphical overview shows heterogeneity (indicated by same cell types in distinct colors) and transition (indicated by single line, black arrows, and different cell types) of all these subpopulations and their capability to adjust their phenotype to the diseased environment. Mono, Monocytes; EC, Endothelial cello; ILC, Innate lymphoid cell; Mo, Macrophage; SMC, Smooth muscle cell; SEM, SMC-derived intermediate; VSMC, Vascular smooth muscle cell; DC, dendritic cell; Mesen, Mesenchymal.

By summarizing the previous single-cell transcriptomics studies, it can be seen that the cytological mechanism of vascular diseases is quite complicated. Each cell subpopulation participates in the occurrence and development of vascular diseases. The explosion of single-cell transcriptomics provides an efficient solution to identify these heterogeneous subpopulations and establish the spatiotemporal dynamic model of vascular biology and pathological cell composition (Figure 1). Specific cell subtype markers are expected to become diagnostic criteria, therapeutic targets, or prognostic indicators.

## Limitations and Future Prospects

Single-cell transcriptomics enables people to truly analyze the entire influence pathway from genes to individual traits from the cellular level in the research of vascular development and vascular diseases, allowing people to better understand how genes affect the entire process of individual traits by affecting the phenotype of cell subpopulations. This is of great significance for precision medicine in vascular medicine. However, with most current sequencing technologies, only one dimension of information can be obtained from a batch of cells and other histological data will be lost. It is necessary to obtain multiple histological information for a single cell, which means that we can establish links between different histological data to better characterize cell function and its internal regulatory processes. Combining several of these dimensions of data into an integrated multi-omics analysis of the same single cell will generate unprecedented knowledge in the field of vascular medicine.

Fresh cell suspensions are required for single-cell sequencing onboarding, so information about the spatial location of cells is destroyed. However, the spatial location of cells plays an important role in the development of disease. To this end, spatial transcriptome technology has been developed, a novel technological breakthrough that reveals the genetic activity in tissues and simultaneously demonstrates the precise regional location of these active signals. The combination of spatial transcriptome and single-cell sequencing technologies has the potential to change our conceptual understanding of vascular biology and disease states and has far-reaching implications for revealing new vascular disease mechanisms and cell type-specific pathways. Further studies of functional heterogeneity and intercellular interactions in human vascular tissues may elucidate disease-related pathophysiological processes and provide a basis for identifying new pathways for vascular disease regulation and developing new therapeutic strategies for early prevention and even reversal of vascular diseases.

## Author Contributions

MF reviewed recent published paper and drafted the manuscript. JS contributed to conception and critically revised manuscript.

## Conflict of Interest

The authors declare that the research was conducted in the absence of any commercial or financial relationships that could be construed as a potential conflict of interest.

## Abbreviations

AA: aortic aneurysm
AAA: abdominal aortic aneurysm
Adv-ECs: adventitia endothelial cells
AECs: arterial endothelial cells
Ao: aortic arch
cMo: conventional monocytes
CyTOF: cytometry by time-of-flight
ECs: endothelial cells
ECM: extracellular matrix
EndMT: endothelial to mesenchymal transition
FMCs: fibroblast-like muscle cells
HECs: hemogenic endothelial cells
HFD: high-fat diet
IL: interleukin
ILCs: innate lymphoid cells
intMo: intermediate monocyte
IPAH: idiopathic pulmonary artery hypertension
PAH: pulmonary artery hypertension
pMo: patrolling monocytes
scRNA-seq: single-cell RNA sequencing
SEM: SMC-derived intermediate
STING: stimulator of interferon genes
TREM2: triggered receptor expressed on myeloid cells 2
VECs: vein endothelial cells
VSMCs: vascular smooth muscle cells
WD: Western diet
WT: wild type.
