# Supplementary material for: Single-Cell Transcriptomics Reveals the Cellular Heterogeneity of Cardiovascular Diseases
Source: Front Cardiovasc Med. 2021 Jun 11;8:643519. doi: 10.3389/fcvm.2021.643519 (PMC8225933; doi:10.3389/fcvm.2021.643519)
Supplement: Supplementary file 1 [file Table_1.DOCX]

***Supplementary File.***

**Supplementary Table 1. Recent reports identified subtypes and functional characteristics of mononuclear phagocytes in vasculature**

| **Species** | **Gender and genotype** | **Tissue** | **Exposure** | **Method** | **Mononuclear phagocytes subtypes** | | | | **Referencr** |
| --- | --- | --- | --- | --- | --- | --- | --- | --- | --- |
|  |  |  |  |  | **Number of clusters (cell type)** | **Name** | **Gene expression** | **Function** |  |
| Mus musculus | Male Ldlr ^-/-^ | Aorta  CD45^+^ | WD 12W | scRNA-seq, FACS | 2 (Macro) | Foamy | CD36, Mertk, Nr1h3, Abca1, Fabp4, Lipa, Mertk | Enhance lipid absorption and excretion | ^1^ |
|  |  |  |  |  |  | Non-Foamy | Ccl2, Ccr2, Abca7, I1b, Nfkbia, Tlr2, Nf- b, IL-17, TLR, Il1, Tnf | Proinflammatory |  |
|  | ApoE^-/-^ | Aorta  CD45^+^ | HFD 12W | CyTOF | 5 (Macro) | Resident 1 | F4/80, CD11b, CD68, CD64, CD206, CD169, CD209b | Regression | ^2^ |
|  |  |  |  |  |  | Resident 2 | F4/80, CD11b, CD68, CD64, CD206, CD169, MHCII |  |  |
|  |  |  |  |  |  | CD11c^+^ | F4/80, CD11b, CD68, CD64, CD44, MHCII, CD206^neg-low^, CD11c | Proinflammatory |  |
|  |  |  |  |  |  | CD206^lo-int^ | F4/80, CD11b, CD68, CD64, CCR2, CD206^low^, CD206^int^ |  |  |
|  |  |  |  |  |  | F4/80^hi^ CD11b^hi^ | F4/80, CD11b, CD68, CD64 | Adventitia Macro |  |
|  | Male Ldlr ^-/-^ /ApoE^-/-^ | Aorta  CD45^+^ | HFD 11W/WD 12W  CHD | scRNA-seq | 3 (Macro) | Res-like | Adgre1, Csf1r, Fcgr1, Cd68, F13a1, Lyve1, Gas6, Ccl24, Ccl9, Folr2, Cbr2, Mrc1, Pf4, Txnip |  | ^3^ |
|  |  |  |  |  |  | Inflammatory | Adgre1, Cd14, Fcgr1, Csf1r, Ly6c2^low^, Cxcl2, Ccl3, Ccl4, Tlr2, Nlrp3, Il1b, Il1a, Cebpb, Egr1, Phlda1, Nfkbiz, Nfkbid, Ier320, Zpf36, Nr4a1, Nfe2l2 | Proinflammatory |  |
|  |  |  |  |  |  | TREM2^hi^ | Adgre1, Csf1r, Fcgr1, Cd68, Trem2, Cd9, Spp1, Hvcn1, Ctsd, Ctsb, Ctsz; | Lipid metabolism, calcification |  |
|  |  |  |  |  | 2 (DC) | Cd209a^+^ | Flt3, Ifi30, Napsa, Itgb7, Syngr2, Clec10a, Ahr, Il1b, Ccr2 | Mono-derived DC |  |
|  |  |  |  |  |  | Cd209a^-^ | Flt3, Ifi30, Napsa, Itgb7, Syngr2, Clec10a, Ahr, Il1b, Ccr7, Fscn1, Cacnb3 | Classic DC |  |
|  | ApoE^-/-^ | Aortic arch CD45^+^ CD11b^+^ F4/80^+^ | WD 16W+ aorta transplantation to WT CD68-GFP | scRNA-seq | Macro | M2 | Apoe, Cd206, Arg1 | M2 Macro derived from Mono | ^4^ |
|  | Male WT  ApoE^-/-^ | Aortic adventitia | CHD | scRNA-seq | 2 (Macro) | Resident | Folr2, Mrc1, Cbr2, Pf4, Sepp1, C1qa, Cxcl12 | Inflammatory | ^5^ |
|  |  |  |  |  |  | Inflammatory | Ms4a6c, Gngt2, Lgals3, Adgre1, F13a1 | Proinflammatory |  |
|  |  |  |  |  | 2 (Mono) | Mono-1 | Clec4d, Xcl2, Il1b, Il1r2 | Atherogenic |  |
|  |  |  |  |  |  | Mono-2 | Anxa1, Wfdc21, Adpgk | Glycolysis |  |
|  | Rank^Cre^Rosa26^eYFP^ | Aortic adventitia CD45^+^ |  | scRNA-seq | 3 | Homeostatory | Adgre1, Itgam, Lyve-1, Stab1, Gas6, Ccr2 | Homeostasis | ^6^ |
|  |  |  |  |  |  | Inflammatory | Adgre1, Itgam, Ly6c, Cxcr4, Cx3cr1, Nr4a1, Il1ß, S100a9 | Inflammatory |  |
|  |  |  |  |  |  | Antigen-presenting | Adgre1, Itgam, H2-Ab1, H2-Aa, Retnla, CD74, Ear2, Mki67 | Antigen presentation |  |
| Homo sapiens | ApoE ^-/-^ | Abdominal aorta | WD 2W + Ang II 4W | scRNA-seq | 2 (Macro) | Ntn1^+^ | Mmp3, Fmo3, F3, Mir143hg, Il18rap, Perp, Ntn1, Ncam1, Ntn4, Angptl7, Esm1, Nov, Efhd1, Cnn1, Cdhr3 | Pro-vascular remodeling | ^7^ |
|  |  |  |  |  |  | Ntn1^-^ | Gdf3, Scd1, CD36, Cidec, Dgat2, Apoc1, Tnmd, Pf4, Mrc1, Adipoq, Hp | Anti-vascular remodeling |  |
|  | Male and Female | ATAA aorta  healthy aorta |  | scRNA-seq | 8 (Macro) | M1-like1 | Tnf, Il1b, Nfkb1, Ccl3l1, Ccl4l2, Ccl4, | Inflammatory | ^8^ |
|  |  |  |  |  |  | M1-like2 | Tnf, Il1b, Nfkb1, Ereg, Areg, Timp1, Vcan | Tissue remodeling, Inflammatory |  |
|  |  |  |  |  |  | M1-like3 | Ets1, Runx2 | Present antigens to CD8 T lymphocytes |  |
|  |  |  |  |  |  | M2-like1 | Mertk, Mrc1, Stab1, Cd163, Pdk4, Stab1, Txnip, Maf | Glucose metabolism, anti-inflammation, phagocytosis |  |
|  |  |  |  |  |  | M2-like2 | Mertk, Mrc1, Stab1, Cd163, C1qa, C1qb, C1qc, Rab13, |  |  |
|  |  |  |  |  |  | M_IFNresponse | Mertk, Mrc1, Stab1, Cd163, Ifi44l, Isg15, Ifit1, Ifitm3, |  |  |
|  |  |  |  |  |  | M_remodeling | Mertk, Mrc1, Stab1, Cd163, Igfbp7, Adirf, Dstn, Tpm2, Mgp, Myl9, Adamts1,  Mmp2, Ctsf | Tissue remodeling |  |
|  |  |  |  |  |  | M_Proliferating | Mertk, Mrc1, Stab1, Cd163, H2afz, Hmgb2, Hmgn2, Tubb, Tuba1b, Stmn, Cks1b |  |  |
|  | Cx3cr1^CreERT2-IRES-YFP/+^Rosa26^fl-tdTomato/+^ | Aortic arch TdTomato^+^CD11b^+^ | AAVmPCSK9+WD 18W/(WD 20W+ ApoB-ASO 2W)+Tamoxifen | scRNA-seq | 10 (Mono) | chemokine^hi^ | Csf1r, CD14, Adgre, CD68, Ccl4, Cxcl2, Ccl3, Ccl2, Tnf, Cxcl1, Cxcl10, Ccl5 | Chemotaxis | ^9^ |
|  |  |  |  |  |  | TREM2^hi^ | Csf1r, CD14, Adgre, CD68, Cxcl16, Atox1, CD72, Glipr1, Trem2, CD9, Lgals3, Spp1, Aldoa | Lipid metabolism, catabolic metabolism and lesion calcification |  |
|  |  |  |  |  |  | NMES1^hi^ | Csf1r, CD14, Adgre, CD68, Cxcl16, Atox1, CD72, Glipr1, NMES1 |  |  |
|  |  |  |  |  |  | IFN signature^hi^ | Csf1r, CD14, Adgre, CD68, IRF7, ISG15, MNDA, IFITM3, Ly6e, Ly6a | Chemotaxis |  |
|  |  |  |  |  |  | CD74^hi^MHC II^hi^ | Csf1r, CD14, Adgre, CD68, CD9, H2-ab1, H2-eb1, H2-aa | Antigen presentation |  |
|  |  |  |  |  |  | DNase1l3^hi^ | Csf1r, CD14, Adgre, CD68, DNase1l3 | interferon response |  |
|  |  |  |  |  |  | Retnla^hi^Ear2^hi^ | Csf1r, CD14, Adgre, CD68, Retnla, Ear2, IL-4 | Chemotaxis |  |
| Mus musculus |  |  |  |  |  | Ebf1^hi^CD79^hi^ | Csf1r, CD14, Adgre, CD68, Ebf1, CD79a |  |  |
|  |  |  |  |  |  | HSP ^hi^ | Csf1r, CD14, Adgre, CD68, Hspa1a, Hspa1b, Stab1, Sepp1 |  |  |
|  |  |  |  |  |  | Stem-like | Csf1r, CD14, Adgre, CD68, Cx3cr1, Stemness, Kit |  |  |
|  | Male 10-week-old C57BL/6J | Infrarenal abdominal aorta | elastase-induced AAA at 7, 14, and 28 days | scRNA-seq | 5 (Mono/Macro) | Mo /MΦ_1 | Cd14，Cd68，Adgre1, H2-Aa, Fcgr1, Pf4, Sepp1 | Phagocytosis, vascular remodelling | ^10^ |
|  |  |  |  |  |  | Mo /MΦ_2 | Cd14，Cd68，Adgre1, H2-Aa, Fcgr1, Ms4a7, Hexb | Blood-derived monocytes |  |
|  |  |  |  |  |  | Mo /MΦ_3 | Cd14，Cd68，Adgre1, H2-Aa, Fcgr1, Thbs1, Plac8 | Mo-derived macrophages |  |
|  |  |  |  |  |  | Mo /MΦ_4 | Cd14，Cd68，Adgre1, H2-Aa, Fcgr1, Ms4a7, Hexb, Phf11b, Irf7 | Mo-derived macrophages |  |
|  |  |  |  |  |  | Mo /MΦ_5 | Cd14，Cd68，Adgre1, H2-Aa, Fcgr1, Birc5, Top2a | Aortic-resident macrophage |  |
|  | Male 12-week-old C57BL/6J | infrarenal abdominal aortas | CaCl2-induced AAA | scRNA-seq | 3 (Macro) | MΦ-1 | Pf4, Lyz2, Mrc1 | Pro-inflammatory macrophage | ^11^ |
|  |  |  |  |  |  | MΦ-2 | Lyz2, Il1b, H2-Ab1 | Inflammatory macrophage |  |
|  |  |  |  |  |  | MΦ-3 | Lyz2, Stmn1, Mki67 | Proliferative macrophage |  |
|  | Female WT | Blood Lin^−^ CD11b^+^CD115^+^ |  | MARS-seq | 4 (Mono) | Ly6C^+^ | Lin^−^, CD11b, CD115, Ly6C^hi^, CD62L^hi^, Lyz1-2, Ccr2, Ly6c2, Mpeg1, Sell, Irf8 | Proliferation | ^12^ |
|  |  |  |  |  |  | Ly6C^int-1^ | Lin^−^, CD11b, CD115, Ly6C^int^, CD62L^int^, Cd74, H2-aa, Ciita, Ccr2, Cd209a | Dendritic cell -like Mono |  |
|  |  |  |  |  |  | Ly6C^int-2^ | Lin^−^, CD11b, CD115, Ly6C^int^, CD62L^int^, Cebpb, Nr4a1, Ccr2, Cd74^int^, H2-aa^int^, Ciita^int^ | Intermediate-Mono |  |
|  |  |  |  |  |  | Ly6C^−^ | Lin^−^, CD11b, CD115, Ly6C^low^, CD62L^low^ , Nr4a1, Cebpb, CD36, Pparg, Itgax, Itgal | Anti- atherogenic |  |
|  | Male and Female Lyn^-/-^ | Blood Lin^−^ CD11b^+^CD115^+^ |  | CyTOF, scRNA-seq | 20 (Mono) | Circulating Mono | 11: CD115, CD11b, Ly6C, CD16, CD32, CD64, CD62L, pTyr, pS6, pSTAT1, CD43, Fcgr4 |  | ^13^ |
|  |  |  |  |  |  | Classical Mono | 6: CD115, CD11b, CD43, FcγRIV, CD274 (PD-L1), CD11c, Sca-1 (Ly6A), pStat3, pp38, IκBα, pErk1/2, pStat5, Ly6C |  |  |
|  |  |  |  |  |  | MHC II^+^ IntMo | 3: CD115, CD11b, MHC II, SIRBa, pp38, IκBα, pERK1/2, pStat1, pAkt, pTry, pStat3, pStat5 |  |  |
| Homo sapiens |  | Healthy blood PBMCs |  | CyTOF | 3 (Mono) | Classical Mono | CD14, CD36, CCR2 |  | ^14^ |
|  |  |  |  |  |  | Intermediate Mono | CD16, HLA-DR^hi^, CD11c, CD14, CCR2, CD36 |  |  |
|  |  |  |  |  |  | Non-classical Mono | CD16, CD11c, HLA-DR^low^ |  |  |
|  | Male and Female | Healthy blood |  | CyTOF, RNA-seq | 8 (Mono) | TREM-1^+^ | CD61, CD9, CD43, CD41, CD14, Trem-1 | Classical Mono | ^15^ |
|  |  |  |  |  |  | CD93^hi^ | CD93, CD11a, CD41, CD9, CD244, CD14 | Classical Mono |  |
|  |  |  |  |  |  | Intermediate Mono | Cx3cr1, CD16, Hla-dr, CD14, Ccr2, CD36, Ctdspl, C1qb, C1qa, Fam60a, Flgas1, Jchain, Hcrt, F13a1, Sparc, Bex3, Gng11, Tim3, CD40, CD81, CD43, CD41, CD9, CD244 |  |  |
|  |  |  |  |  |  | Slan^+^CD9^+^ | Cx3cr1, CD16, CD61, CD9, Slan, B3galt2, CD41, CD43, CD244, Cxcr6 | Cell adhesion, platelet binding, exocytosis |  |
|  |  |  |  |  |  | Slan^+^CD9^-^ | Cx3cr1, CD16, Slan, B3galt2, CD43, CD244, Cxcr6 |  |  |
|  |  |  |  |  |  | CD93^low^ | CD36, CD64, CD14, CD163, Hla-dr ^low^, CD86^low^, CD11a^low^ , CD11c^low^ | Classical Mono |  |
|  |  |  |  |  |  | Slan^-^ | Cx3cr1, CD16, C1qb, C1qa, Fam60a, Flgas1, Jchain, Ttpa, Eno1-as1, Hcrt, CD40, CD244 | Non-classical Mono |  |
|  |  |  |  |  |  | IgE^+^ | IgE, CD1c, Tim3, CD81, CD244, CD14 | Classical Mono |  |

A summarize of recent reports identified subtypes and functional characteristics of mononuclear phagocytes in vasculature. MARS-seq, Massively parallel single-cell RNA-seq; PBMCs, Peripheral blood mononuclear cells. Ang II, Angiotensin II. CyTOF, Cytometry by Time-Of-Flight; WT, Wild-type; WD, Western diet; W, Weeks; CHD, Chow diet; HFD, High fat diet; Macro, Macrophages; Mono, Monocytes; DC, dendritic cells; ATAA, Ascending thoracic aortic aneurysm.

1. Kim K, Shim D, Lee JS, Zaitsev K, Williams JW, Kim KW, Jang MY, Seok Jang H, Yun TJ, Lee SH, et al. (2018). Transcriptome Analysis Reveals Nonfoamy Rather Than Foamy Plaque Macrophages Are Proinflammatory in Atherosclerotic Murine Models*.* *Circ Res*. 123:1127-1142. doi: 10.1161/circresaha.118.312804.

2. Cole JE, Park I, Ahern DJ, Kassiteridi C, Danso Abeam D, Goddard ME, Green P, Maffia P, and Monaco C. (2018). Immune cell census in murine atherosclerosis: cytometry by time of flight illuminates vascular myeloid cell diversity*.* *Cardiovasc Res*. 114:1360-1371. doi: 10.1093/cvr/cvy109.

3. Cochain C, Vafadarnejad E, Arampatzi P, Pelisek J, Winkels H, Ley K, Wolf D, Saliba AE, and Zernecke A. (2018). Single-Cell RNA-Seq Reveals the Transcriptional Landscape and Heterogeneity of Aortic Macrophages in Murine Atherosclerosis*.* *Circ Res*. 122:1661-1674. doi: 10.1161/circresaha.117.312509.

4. Rahman K, Vengrenyuk Y, Ramsey SA, Vila NR, Girgis NM, Liu J, Gusarova V, Gromada J, Weinstock A, Moore KJ, et al. (2017). Inflammatory Ly6Chi monocytes and their conversion to M2 macrophages drive atherosclerosis regression*.* *J Clin Invest*. 127:2904-2915. doi: 10.1172/jci75005.

5. Gu W, Ni Z, Tan YQ, Deng J, Zhang SJ, Lv ZC, Wang XJ, Chen T, Zhang Z, Hu Y, et al. (2019). Adventitial Cell Atlas of wt (Wild Type) and ApoE (Apolipoprotein E)-Deficient Mice Defined by Single-Cell RNA Sequencing*.* *Arterioscler Thromb Vasc Biol*. 39:1055-1071. doi: 10.1161/atvbaha.119.312399.

6. Weinberger T, Esfandyari D, Messerer D, Percin G, Schleifer C, Thaler R, Liu L, Stremmel C, Schneider V, Vagnozzi RJ, et al. (2020). Ontogeny of arterial macrophages defines their functions in homeostasis and inflammation*.* *Nat Commun*. 11:4549. doi: 10.1038/s41467-020-18287-x.

7. Hadi T, Boytard L, Silvestro M, Alebrahim D, Jacob S, Feinstein J, Barone K, Spiro W, Hutchison S, Simon R, et al. (2018). Macrophage-derived netrin-1 promotes abdominal aortic aneurysm formation by activating MMP3 in vascular smooth muscle cells*.* *Nat Commun*. 9:5022. doi: 10.1038/s41467-018-07495-1.

8. Li Y, Ren P, Dawson A, Vasquez HG, Ageedi W, Zhang C, Luo W, Chen R, Li Y, Kim S, et al. (2020). Single-Cell Transcriptome Analysis Reveals Dynamic Cell Populations and Differential Gene Expression Patterns in Control and Aneurysmal Human Aortic Tissue*.* *Circulation*. 142:1374-1388. doi: 10.1161/circulationaha.120.046528.

9. Lin JD, Nishi H, Poles J, Niu X, McCauley C, Rahman K, Brown EJ, Yeung ST, Vozhilla N, Weinstock A, et al. (2019). Single-cell analysis of fate-mapped macrophages reveals heterogeneity, including stem-like properties, during atherosclerosis progression and regression*.* *JCI Insight*. 4. doi: 10.1172/jci.insight.124574.

10. Zhao G, Lu H, Chang Z, Zhao Y, Zhu T, Chang L, Guo Y, Garcia-Barrio MT, Chen YE, and Zhang J. (2020). Single cell RNA sequencing reveals the cellular heterogeneity of aneurysmal infrarenal abdominal aorta*.* *Cardiovasc Res*. doi: 10.1093/cvr/cvaa214.

11. Yang H, Zhou T, Stranz A, DeRoo E, and Liu B. (2021). Single-Cell RNA Sequencing Reveals Heterogeneity of Vascular Cells in Early Stage Murine Abdominal Aortic Aneurysm-Brief Report*.* *Arterioscler Thromb Vasc Biol*. 41:1158-1166. doi: 10.1161/atvbaha.120.315607.

12. Mildner A, Schönheit J, Giladi A, David E, Lara-Astiaso D, Lorenzo-Vivas E, Paul F, Chappell-Maor L, Priller J, Leutz A, et al. (2017). Genomic Characterization of Murine Monocytes Reveals C/EBPβ Transcription Factor Dependence of Ly6C(-) Cells*.* *Immunity*. 46:849-862.e847. doi: 10.1016/j.immuni.2017.04.018.

13. Roberts ME, Barvalia M, Silva J, Cederberg RA, Chu W, Wong A, Tai DC, Chen S, Matos I, Priatel JJ, et al. (2020). Deep Phenotyping by Mass Cytometry and Single-Cell RNA-Sequencing Reveals LYN-Regulated Signaling Profiles Underlying Monocyte Subset Heterogeneity and Lifespan*.* *Circ Res*. 126:e61-e79. doi: 10.1161/circresaha.119.315708.

14. Thomas GD, Hamers AAJ, Nakao C, Marcovecchio P, Taylor AM, McSkimming C, Nguyen AT, McNamara CA, and Hedrick CC. (2017). Human Blood Monocyte Subsets: A New Gating Strategy Defined Using Cell Surface Markers Identified by Mass Cytometry*.* *Arterioscler Thromb Vasc Biol*. 37:1548-1558. doi: 10.1161/atvbaha.117.309145.

15. Hamers AAJ, Dinh HQ, Thomas GD, Marcovecchio P, Blatchley A, Nakao CS, Kim C, McSkimming C, Taylor AM, Nguyen AT, et al. (2019). Human Monocyte Heterogeneity as Revealed by High-Dimensional Mass Cytometry*.* *Arterioscler Thromb Vasc Biol*. 39:25-36. doi: 10.1161/atvbaha.118.311022.
